# Kidney Disease Segmentation and Classification Using Firefly Sigma Seeker and MagWeight Rank Techniques

**DOI:** 10.3390/bioengineering12040350

**Published:** 2025-03-28

**Authors:** Dilovan Asaad Zebari

**Affiliations:** 1 Computer Science, College of Science, Nawroz University, Duhok 42001, Kurdistan Region, Iraq; dilovan.majeed@nawroz.edu.com; 2 Faculty of Computing and Information Technology, Sohar University, Sohar 311, Oman

**Keywords:** early detection, Firefly Sigma Seeker, MagWeight Rank, optimization techniques, parallel convolutional layers, Multi-Stream Neural Network (MSNN)

## Abstract

Deep learning models possess the ability to precisely analyze medical images such as MRI, CT scans, and ultrasound images. This automated diagnostic process facilitates the early detection of kidney disease by identifying any abnormalities or signs of disease. Consequently, it allows for timely intervention and treatment, while also reducing the need for manual interpretation by radiologists or clinicians. As a result, the diagnosis process is expedited, leading to improved efficiency in healthcare. The proposed technique focuses on enhancing parallel convolutional layer architectures in kidney disease segmentation through the utilization of advanced optimization techniques. This approach integrates Firefly Sigma Seeker and MagWeight Rank methodologies into the design of these architectures. The Firefly Sigma Seeker methodology dynamically adjusts key parameters related to standard deviation during training to enable early stopping in the initial phase. Subsequently, MagWeight Rank optimizes parameter weighting and ranking within the architecture to prune less important weights, thereby reducing computational time and overfitting. By leveraging these techniques, the parallel convolutional layers are specifically tailored for kidney disease segmentation tasks. Finally, the Multi-Stream Neural Network (MSNN) efficiently classifies kidney disease. Through extensive experimentation and evaluation on kidney disease segmentation datasets, a comparative analysis of different architectures was conducted in terms of segmentation accuracy, computational efficiency, and scalability. The proposed framework achieves optimal segmentation performance, with an accuracy of 98.2%, a minimized loss of 0.1, a reduced computational time of 15 min and 4 s, and successfully avoids overfitting.

## 1. Introduction

Kidney disease is a significant global health concern that impacts a large population and can have severe consequences if not detected and treated early. Timely identification and diagnosis of kidney disease are crucial for initiating prompt interventions and enhancing patient outcomes. Medical imaging plays a vital role in identifying and diagnosing kidney disease by providing valuable insights into the structural and functional abnormalities of the kidneys. It is very crucial to segment and classify kidney structures and anomalies so as to enable diagnosis and planning of treatment as well as monitoring of the progress of the diseases. For instance, the Bosniak classification system is used when dealing with renal cysts’ imaging to determine the likelihood of malignancy. Similarly, the classification of chronic kidney disease (CKD) into stages based on glomerular filtration rate (GFR) and albuminuria levels is essential for determining appropriate management strategies.

Recently, CNNs have emerged as powerful tools for predicting, segmenting, and classifying kidney diseases using medical imaging data. These techniques employ intricate neural network architectures to automatically extract features from medical images, enabling accurate predictions and classifications. These approaches have shown promising results in various medical imaging tasks, including the prediction, segmentation, and classification of kidney diseases. Deep learning methods commonly involve the utilization of Convolutional Neural Networks (CNNs) or other deep learning architectures for predicting, segmenting, and classifying kidney disease [1]. CNNs are highly effective in analyzing medical images because of their capability to capture spatial hierarchies of features. When focusing on segmenting kidney disease, CNN-based segmentation models can precisely identify regions of interest within medical images, like the kidneys or lesions, facilitating accurate diagnosis and treatment planning [2]. These models often integrate encoder–decoder structures and skip connections, such as U-Net, FCN, or PSPNet, to capture both local and global features [3]. In classification tasks, deep learning models are trained to classify medical images into various classes based on the presence or severity of kidney disease [4]. These models extract unique features from the images and make predictions using softmax or sigmoid output layers. Commonly utilized architectures for classification tasks include CNNs, ResNets, or DenseNets, which excel in distinguishing between healthy and diseased kidneys or different disease stages [5].

The advancements achieved in deep learning-based techniques for predicting, segmenting, and categorizing kidney diseases have significantly improved diagnostic capabilities. Nevertheless, there exist persistent barriers that impede their widespread adoption and efficacy in clinical settings. A primary obstacle is the scarcity of labeled medical imaging data, coupled with concerns regarding data quality and variability. These factors complicate the creation of robust and universally applicable models. Manual methods are lengthy and evoke inter observer variation and, therefore, there is inconsistency in the diagnosis. Most of the conventional methods of mechanization incorporate a simple form of feature extraction, thus are not very efficient. Furthermore, deep learning models lack transparency, presenting difficulties for healthcare providers who rely on accurate predictions for informed decision-making. Additionally, it is problematic to extend models trained on specific populations or imaging methods to diverse patient groups and clinical environments, limiting their practical utility. Moreover, the computational requirements of intricate deep learning structures hinder their feasible implementation in healthcare settings with limited resources. These limitations result in high dependence from large, annotated datasets, while these are limited in medical imaging. As such, this may lead to the overfitting of certain databases, thus limiting generalization of methodology to the other populations and imaging techniques. Because of the lack of interpretability, clinicians will not trust or use these models reliably in their practice. That is why its implementation in most healthcare facilities is already a challenge marked by having high computational requirements. To address these challenges, continuous research endeavors are essential to devise deep learning methodologies that are efficient with data and that are interpretable and adaptable. The key findings of the study are elaborated upon below.

1. The proposed work combines two advanced optimization techniques, Firefly Sigma Seeker and MagWeight Rank, to develop parallel convolutional layer architectures for the segmentation and classification of kidney disease.
2. The combination of the two advanced optimization techniques, Firefly Sigma Seeker and MagWeight Rank, is proposed in this work to develop parallel convolutional layer architectures for the segmentation and classification of kidney disease.
3. By incorporating these techniques, the proposed framework achieves superior segmentation performance compared to existing methods.
4. These techniques enable the dynamic adjustment of key parameters related to standard deviation during training and optimize parameter weighting and ranking within the architecture, resulting in enhanced accuracy in kidney disease segmentation.
5. Furthermore, the integration of Firefly Sigma Seeker and MagWeight Rank enhances the utilization of computational resources by reducing overfitting and computational time.
6. This ensures that deep learning models can be effectively trained and deployed in healthcare environments with limited resources.

The rest of this document is organized as follows. Section 2 reviews related literature on kidney disease segmentation and classification. Proceed to Section 3 to read the discussion on different approaches to classifying kidney disease. Section 4 describes the experimental methods used, and Section 5 summarizes the results and conclusions of the study.

## 2. Related Works

Kriplani et al. conducted a study that focused on the application of artificial neural networks (ANNs), in predicting chronic kidney diseases (CKD). The study discussed the steps involved in data preprocessing, feature selection, and training a deep neural network structure using clinical parameters and medical imaging data. The results demonstrate the model’s performance measures, such as accuracy and area under the ROC curve, highlighting its effectiveness in predicting CKD. This research contributes to the improvement of early detection and intervention strategies for CKD by utilizing CNN methods, emphasizing their potential in enhancing patient outcomes and healthcare management [5]. Ma et al. employed a heterogeneous modified Artificial Neural Network (ANN) to detect and diagnose chronic kidney disease (CKD). The study involved the development and training of a customized ANN architecture capable of handling various types of data inputs, including clinical metrics, laboratory findings, and medical imaging data. The results demonstrate the efficacy of the model in accurately identifying and diagnosing CKD, thereby improving automated diagnostic tools for enhanced patient care and healthcare management [6]. Kuo et al.’s research was on utilizing a deep model with ultrasound-based kidney imaging to automate the prediction and classification of kidney function. The study aims to develop deep learning models that are trained on ultrasound images to accurately predict kidney function and categorize kidney diseases. The results of this study demonstrated the effectiveness of these models in accurately predicting kidney function and categorizing kidney diseases, which could lead to advancements in automated diagnostic tools for kidney-related conditions [7].

Debal et al. investigate a method using Support Vector Machines (SVM), Decision Tree, and Random forests in the prediction of chronic kidney disease (CKD). The study involved the utilization of various machine learning algorithms on clinical data, encompassing demographic information, laboratory results, and medical records, to develop predictive models for CKD. Results demonstrated the efficacy of these models in accurately forecasting the onset or progression of CKD, offering valuable insights into the potential advantages of machine learning in early disease detection and risk assessment [8]. Chittora et al. carried out an extensive investigation based on Logistic Regression, Random forests, SVM, KNN, and Naive Bayes; in addition to using Correlation-based Feature Selection (CFS) and Principal Component Analysis (PCA) in predicting chronic kidney disease (CKD). The study involved the use of different machine learning algorithms to analyze a diverse range of clinical data, including patient demographics, medical history, and laboratory results. The main aim was to develop predictive models for the early detection and progression of CKD. The findings demonstrated the efficacy of these models in accurately predicting CKD, thus providing valuable insights into the potential of machine learning approaches for disease detection and risk assessment in relation to kidney health [9].

The process of chronic kidney disease (CKD) detection through machine learning involves collecting demographic and clinical data, handling missing values and outliers, choosing relevant features, selecting appropriate models, and training and evaluating the models using accuracy and AUC-ROC metrics, adjusting hyperparameters for better performance, validating model accuracy on independent datasets, interpreting model predictions, and potentially deploying the model in clinical settings for early CKD identification and risk assessment [10]. Akter et al. performed an extensive analysis on deep learning models to predict and detect the risk of chronic kidney disease (CKD) in its early stages. The study involved evaluating different deep learning structures trained on diverse datasets that included patient demographics, medical history, and laboratory findings. The findings demonstrate the effectiveness of these models in accurately forecasting the onset or progression of CKD [11]. Singh et al. presented a new deep neural network methodology designed to quickly identify and predict chronic kidney disease. The study involved developing and evaluating a deep learning model trained on a broad range of clinical data, such as demographic information, medical records, and laboratory findings to accurately predict the onset or progression of CKD. The results demonstrated the effectiveness of the deep neural network in identifying individuals at risk of CKD at an early stage, highlighting its potential as a valuable tool for improving patient outcomes through timely intervention and effective management of kidney health [12].

Saif et al. present Deep-kidney, a robust deep learning framework specifically created for the prediction of chronic kidney disease (CKD). Here, an attempt has been made to utilize more sophisticated models, among them CNN, LSTM, and LSTM-BLSTM, which have been trained from different clinical databases containing information from patients’ lab results and their history. The primary aim is to precisely forecast the likelihood of CKD occurrence or progression. The methodology involves the collection of a wide range of clinical datasets, including patient demographics, medical history, and laboratory results. Subsequently, the data are preprocessed to handle missing values and outliers, and relevant features are selected. Advanced deep learning models are then designed and trained using this comprehensive approach. The performance of the models is assessed using metrics such as accuracy and AUC-ROC, and their validity is confirmed using independent datasets. This comprehensive approach ensures the development of an efficient deep learning framework for the prediction of chronic kidney disease. The framework possesses the capability to provide accurate and timely predictions, thereby greatly aiding in early intervention and the management of kidney health [13].

Goel et al. introduce a novel deep learning model specifically designed for the segmentation of kidneys in magnetic resonance imaging (MRI) scans of individuals diagnosed with polycystic kidney disease (PKD). This advanced model has been trained on a dataset of PKD MRI scans using sophisticated 3D U-Net model, enabling it to accurately segment and outline kidney structures. The outcomes of this research demonstrated the remarkable efficacy of the deployed deep learning model in automating and precisely delineating kidney segmentation. Consequently, this model will play a vital role in aiding the diagnosis and monitoring of PKD patients through MRI [14]. Hsiao et al. have introduced an innovative system for kidney segmentation. This system harnesses the power of deep model, specifically incorporating efficient Feature Pyramid Networks (FPNs). Its primary objective is to accurately segment kidney structures from CT scans, offering a dependable and automated approach. The anticipated research outcomes aim to demonstrate the effectiveness of this deep learning system in improving segmentation accuracy and efficiency. Consequently, it greatly aids in clinical tasks such as disease diagnosis and treatment planning [15]. Couteaux et al. introduced a method for segmenting the kidney cortex in Two-Dimensional (2D) Computed Tomography (CT) images. Utilizing U-Net models and ensemble aggregation techniques, they successfully achieved this objective. This strategy entails employing several U-Net architectures to segment the kidney cortex from CT scans. Through the fusion of predictions from different models via ensemble aggregation, the precision of segmentation is enhanced. The outcomes of the research demonstrate the efficiency of this approach in precisely outlining the kidney cortex, thus offering significant assistance in diagnostic imaging for different kidney-related ailments [16].

Multitask and multifold machine learning techniques demonstrate strong potential to develop improved diagnosis systems for kidney diseases along with classification strategies. Advanced AMMW image technology enables security teams to detect hidden objects through real-time scans, which operate non-contact at high throughputs for surveillance of large public facilities including railway stations and airports [17]. The addition of an attention mechanism enables better suppression of noise and target prioritization, which improves the image processing of concealed objects in AMMW images. The thermal imager detects thermal traces in an invisible form while analysis of infrared heat patterns extracts data about identify and time relation within the trace. A joint deep convolutional neural network framework called MTLHand simultaneously handles the heat trace identification challenge with the heat trace departure time prediction challenge to boost recognition performance. The model develops invariant representations of thermal palmprint identities, which improve identity recognition accuracy by capturing palmprint data changes [18]. The deep soft threshold feature separation (DSTFS) network has improved identity recognition and time estimation accuracy by implementing a framework that divides identity features from time features [19]. Multi-input along with multi-job learning approaches demonstrate great potential in bringing together multiple imaging techniques with targeted evaluation methods for improving kidney disease diagnosis in clinical settings.

Ensemble learning also helps to reduce the variance of the predictions and improves feature detection through the utilization of higher quality and an increased number of segmentation maps from several U-Nets. In contrast to developing a single U-Net or FCN model, the ensemble algorithm lowers the risk of overfitting and enhances the stability of the model, which results in a superior performance of the algorithm proposed in this paper in terms of tumor segmentation. In a study by Couteaux et al. concerning the use of 3D convolutional neural networks in the segmentation of the kidney cortex, it was established that the technique enhances the accuracy of the identification of kidney diseases. The summary of the literature survey is displayed in Table 1.

## 3. Proposed Method

In the context of our research, we applied a set of operations to the medical images, which facilitated improvement in their quality and standardization. In addition to that, clean data were taken after first eliminating any records that could contain missing or corrupted data from the dataset. In the analysis, cases of missing data were handled through a process known as imputation, where data were assumed to have some particular value depending on the existing values in a particular record. In order to enhance the images to be standardized, we conducted min–max normalization aiming at A pixel intensities normalization across the images. This process reduces fluctuations and makes the images more consistent; that is, images captured in different settings are put under equal measures. Further, to increase the size of the data, specific operations were employed for data augmentation including rotations, flipping, and scaling. These augmentations brought variability, helping to avoid overfitting and increasing the model’s ability to perform well in unseen, new data.

This study utilized multiple regularization approaches to enhance both generalization capability and unseen data handling capabilities in our model. Dropout and L2 regularization served as regularization methods to suppress overfitting through their implementation. These techniques enable data augmentation to build a model with excellent performance along with effective operation on multiple datasets.

The integration of Pyramid Scene Parsing Network (PSPNet) with Firefly Sigma Seeker and MagWeight Rank techniques for kidney disease segmentation and classification signifies a progressive method in medical image analysis. PSPNet, an innovative convolutional neural network architecture, demonstrates exceptional proficiency in capturing contextual information at various scales, a vital aspect for accurately segmenting intricate structures, such as kidneys from medical images. Through the incorporation of PSPNet into the suggested framework, the model acquires the capability to effectively process complex kidney images, guaranteeing precise segmentation outcomes [16]. This work used four layers, each of which is a convolution layer with a kernel size of 3 × 3, a stride of 1, and ‘Same’ padding. Every convolutional layer is also passed through an activation function ReLU to enable the non-linear transformation. Adopting a basic model of medical image segmentation, the architecture of the system has been designed to be efficient in terms of segmentation accuracy.

Firefly Sigma Seeker brings a distinctive element to the approach by dynamically modifying crucial parameters, such as the threshold value, throughout training according to the standard deviation of the validation loss. This adaptive feature enables early termination in the early stages of training, averting overfitting and guaranteeing the model’s ability to generalize. Moreover, Firefly Optimization boosts the optimization procedure by directing the quest for ideal threshold values within the model’s hyperparameter space, thereby enhancing the reliability and effectiveness of segmentation and classification tasks [20]. This way, the threshold value is updated dynamically according to the standard deviation of the validation loss so as to enable an early stopping to thereby avoid overfitting.

MagWeight Rank is an advanced technique that enhances the performance of neural networks by selectively removing less important weights, resulting in reduced computational overhead. By prioritizing important connections and features, MagWeight Rank optimizes the model architecture, improving computational efficiency and enabling faster inference times. This technique is particularly beneficial in medical imaging applications where computational resources may be limited, but accuracy and speed are crucial for timely diagnosis and treatment decisions. Any weight lower in absolute value than a certain value, calculated by the mean absolute value of weights in the layer, is pruned for increasing the efficiency of the model.

FSS along with MWR is used as optimization techniques in order to improve the performance of the model. The FSS utilizes the procedure on how fireflies operate in finding mates, a mechanism FSS adapts in order to search for the best features in a high dimensional feature space. MWR, contrary to this, assigns some weights to various features and this can enhance the key features for even higher accuracy performance. MWR can minimize computational cost since it deals with a reduction in the dimensionality of the feature space by emphasizing only essential features. These techniques help in fast convergence and high accuracy of the division in the process. This leads to more efficient training time, which reduces computational cost and high accuracy in segmentation.

When integrated with Firefly Sigma Seeker and MagWeight Rank, the model achieves both weight optimization for efficiency and stable convergence capabilities. These techniques maintain generalization through the dynamic threshold adjustment, which combines with weight pruning for less significant weights. Early stopping mechanism in Firefly Sigma Seeker stops training cycles before they become excessive to ensure both model convergence and new data generalization. Gradual convergence stability depends on a model loss function, which utilizes gradient descent optimization for its minimization process. The model achieves convergence when its loss function automatically decreases during training. Firefly Sigma Seeker enables early stopping of model training because it helps users find the optimal stopping point that stops overfitting. Through MagWeight Rank-based pruning, researchers can keep the most vital features which create performance enhancements and permit information generalization across different datasets.

The integration of these methods creates a comprehensive system for segmenting and categorizing kidney diseases, effectively addressing challenges such as limited data availability, computational complexity, and model interpretability. By utilizing PSPNet, Firefly Sigma Seeker, and MagWeight Rank, the proposed approach achieves exceptional results in accurately detecting and classifying kidney abnormalities from medical images. Moreover, the framework’s flexibility and efficiency make it highly suitable for implementation in real clinical environments, where prompt and accurate diagnosis is essential for patient care and treatment.

(1) $$z\left(l\right)=W\left(l\right)\times \left(l-1\right)+b\left(l\right)$$

Equation (1) represents the linear transformation carried out at layer *l* within a neural network. In this context, $W\left(l\right)\;$symbolizes the weight matrix, *a*(*l* − 1) denotes the output of the preceding layer, and $b\left(l\right)\;$stands for the bias vector. This operation merges inputs from the previous layer with their respective weights, incorporating a bias component.

(2) $$a\left(l\right)=ReLU\left(z\left(l\right)\right)$$

The computation of layer *l*’s activation is determined by Equation (2), utilizing the Rectified Linear Unit (ReLU) activation function. The variable $z\left(l\right)\;$represents the output of the linear transformation at layer *l*, while the ReLU function performs an element-wise operation by setting negative values to zero and preserving positive values.

(3) $$a\left(l\right)=MaxPool\left(a\left(l-1\right)\right)$$

Equation (3) is employed to conduct max-pooling on the output of the preceding layer *a*(*l* − 1). This process decreases the spatial dimensions of the input tensor by choosing the maximum value within each pooling region, thus preserving crucial features.

(4) $$\sigma_{loss}=\sqrt{\frac{1}{N}}\sum_{i=1}^{N}\left(loss-\bar{loss}\right)$$

The dispersion or variability of the *loss* values from their mean $\bar{loss}$ is quantified by calculating the standard deviation $\sigma_{loss}$ using Equation (4) for a batch of size *N*.

(5) θ=σ×σloss

The threshold θ is determined by multiplying the standard deviation *σ* loss, as shown in Equation (5). This threshold is utilized to implement early stopping in the training process, which is terminated if the loss decrease is less than this threshold value.

(6) $$mean\left(W\right)=n1\sum_{i=1}^{n}|wi|$$

Equation (6) denotes the mean of the absolute values of the weights in a specific layer, referred to as mean (*W*). It is computed by adding up all the absolute values of the weights and then dividing by the total count of weights in that layer. This formula provides important insights into the average weight magnitude within the network, which is helpful in establishing the pruning threshold.

(7) $$Pruning\;Threshold=\alpha \times mean\left(W\right)$$

Equation (7) calculates the pruning threshold, referred to as Pruning Threshold, by multiplying the average of the weights by a hyperparameter α. This critical value is essential for detecting less important weights in the network that can be pruned, with weights exceeding the threshold being kept and those falling below it being pruned.

(8) $$Seg\;Loss=-\frac{1}{N}\sum_{i=1}^{N}\sum_{c=1}^{C}yi,clog\left(pi,c\right)$$

Equation (8) denotes the segmentation loss, referred to as Seg Loss, which is computed by adding together the negative logarithm of the forecasted probabilities for every sample and class, in accordance with the true labels. This particular loss function measures the difference between the predicted segmentation and the real ground truth.

(9) Total Loss=Seg Loss+Class Loss

Equation (9), denoted as Total Loss, is the aggregate of the segmentation loss (Seg Loss) and the classification loss (Class Loss). The classification loss may include diverse supplementary losses associated with the classification task, such as cross-entropy loss or mean squared error.

(10) wt+1=wt−η⋅∇Total Loss

The weight update step is depicted by Equation (10), where wt + 1 represents the updated weights, wt denotes the current weights, η is the learning rate, and ∇Total Loss signifies the gradient of the total loss function.

(11) θt+1=θt−ηθ⋅∇Total Loss

The update step for the pruning threshold (θ) using gradient descent during the optimization process is represented by Equation (11) · ∇Total Loss. In this equation, θt and θt + 1 denote the threshold at time *t* and *t* + 1, respectively, ηθ is the learning rate for the threshold, and ∇Total Loss is the gradient of the total loss with respect to the threshold. This equation updates the pruning threshold to control the magnitude of weight pruning based on the gradient of the total loss.

(12) $$xt+1=xt+\beta \cdot attractiveness\left(xi,xj\right)+\gamma \cdot randomness()$$

Equation (12) represents the update step for the positions (x) of the fireflies in the Firefly Algorithm. It utilizes the variables xt and xt + 1 to denote the positions of fireflies at time *t* and *t* + 1, respectively. The scaling factors *β* and *γ* are also incorporated in this equation. Additionally, the attractiveness (xi,xj) signifies the level of attractiveness between fireflies *i* and *j*. By employing this equation, the positions of fireflies are adjusted, taking into account their attractiveness and randomness. This enables the algorithm to explore and find optimal solutions.

(13) $$At+1=At\cdot exp\left(-\gamma \cdot distance\left(xi,xj\right)\right)$$

The Formula (13) in the Firefly Algorithm is used to update the attractiveness (*A*) between fireflies. It involves the scaling factor *γ*, the attractiveness values At and At + 1 at times *t* and *t* + 1, and the distance between fireflies *i* and *j* denoted by distance (xi,xj). This equation adjusts the attractiveness between fireflies according to their distances.

(14) $$Accuracy=\frac{Correct\;Predictions}{Total\;Predictions}$$

In Formula (14), the term Correct Predictions represents the number of predictions that the model correctly classified or segmented, while “Total Predictions” indicates the total number of predictions produced by the model. By dividing the accurate predictions by the total predictions, we calculate the model’s accuracy, a crucial performance metric for evaluating the model’s effectiveness in classifying or segmenting kidney diseases.

(15) Total Loss=Seg Loss+Class Loss+Auxiliary Loss

Equation (15) illustrates the computation of the overall loss experienced by the model throughout the training process. This total loss is calculated by summing up the segmentation loss, classification loss, and any other supplementary loss components, like regularization terms or auxiliary objectives. The segmentation loss evaluates the difference between the predicted segmentations and the actual ground truth segmentations, whereas the classification loss measures the distinction between the predicted class labels and the true class labels. Through minimizing the total loss, the model’s ability to accurately classify and segment kidney diseases can be enhanced. Algorithm 1 presents the step-by-step methodology for integrating Firefly Sigma Seeker and MagWeight Rank techniques into the segmentation model.
**Algorithm 1.** Pseudocode for Firefly Sigma Seeker and MagWeight Rank TechniquesInitialize model parameters and hyperparameters:-learning_rate (Learning rate)-batch_size (Batch size)-num_epochs (Number of epochs)-alpha (Pruning threshold)-beta (Firefly attractiveness parameter)-gamma (Firefly randomness parameter)-theta (MagWeight Rank parameter)data = load_data() train_loader, val_loader, test_loader = split_data(data, batch_size) segmentation_loss = SegmentationLoss() classification_loss = ClassificationLoss() for epoch in range(epochs): total_loss = 0.0 correct_predictions = 0 total_predictions = 0 batch in train_loader is as follows: seg_predictions, class_predictions = model(batch[‘images’]) seg_loss_value = segmentation_loss(seg_predictions, batch[‘seg_labels’]) class_loss_value = classification_loss(class_predictions, batch[‘class_labels’]) total_loss_value = seg_loss_value + class_loss_value optimizer.zero_grad() total_loss_value.backward() optimizer.step() total_loss += total_loss_value.item() total_predictions += batch_size accuracy_rate = correct_predictions/total_predictions

**Figure 1:**
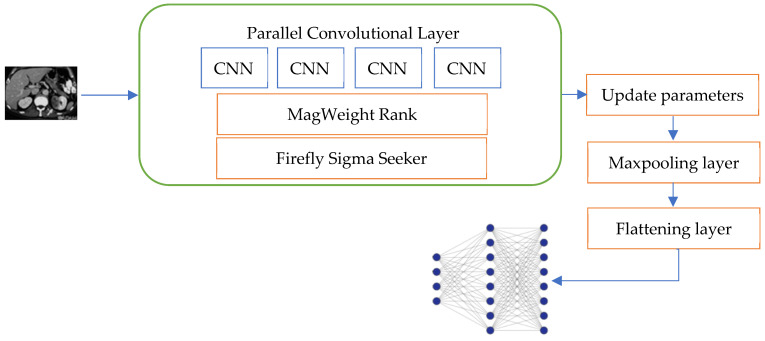
An architecture of proposed optimization in single stream CNN.

The improved single streamline CNN design employs various optimization measures to up its ability to carry out tasks such as kidney disease segmentation and classification, as depicted in Figure 1 below. The architecture can also adapt various parameters and set different weights during the training process, thereby making use of the Firefly Sigma Seeker and MagWeight Rank methods. These techniques help the model to increase the capacity of data, as well as to improve sensitivity to features that are important for accurate prediction. In incorporating these optimization methods, the single stream CNN would enhance accuracy and efficiency in the processing of the medical images for kidney disease analysis. Moreover, the architecture’s design also focuses on the strategized processing of medical image data, thus making it accommodative to the complexity involved in the managing of such information. As a result of the flow of information and the ability of the CNN to have a single stream, input processing provides an efficient stage of deriving features from inputs in addition to reducing the computational demand. It also improves both the parallelism and the practicality of the model and its ability to be applied in real medical imaging tasks. Hence, it can be concluded that the optimality of the single-stream CNN architecture proposed in this paper represents an improvement and innovation in the field of medical image analysis and the diagnosis of kidney diseases.

**Figure 2:**
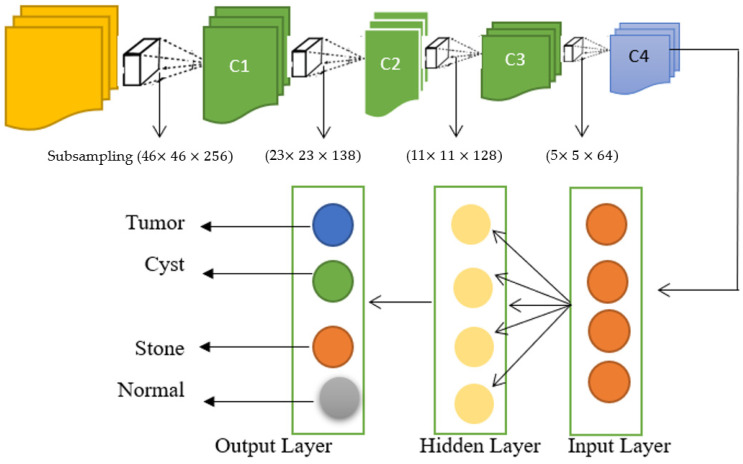
An architecture of Multi stream CNN after MagWeight rank and Firefly Sigma Seeker.

Figure 2 illustrates the structure of a Multi-Stream Convolutional Neural Network (CNN), which highlights the implementation of MagWeight Rank and Firefly Sigma Seeker optimization techniques. The diagram demonstrates the transformation of feature maps through subsampling layers, starting from an input layer with dimensions of 46 × 46 × 256 and progressing to subsequent layers with reduced dimensions of 23 × 23 × 138, 11 × 11 × 128, and finally 5 × 5 × 64. These dimensions represent the varying sizes of the feature maps at different stages of processing within the network. By integrating MagWeight Rank and Firefly Sigma Seeker, the model effectively optimizes parameter weights and dynamically adjusts learning rates, thereby improving its ability to efficiently process medical image data for tasks such as kidney disease segmentation and classification.

The architecture of the Multi-Stream Neural Network (MSNN) consists of multiple stream networks that analyze diverse features or aspects of the data. These streams gather different information in parallel, which are then fused in order to enhance the segmentation performance. This improves the performance of the model since it can analyze more features of the image, capturing a wider range of features. The MSNN has two pathways for processing different features of the inputs and enhances the kidney segmentation performance of the network. The first one is an end-to-end fully convolutional network known as U-Net, and the second is called DeepLabv3+, which makes use of atrous convolutions (also referred to as Dilated convolutions) for capturing multi scale features. Generative Adversarial Networks (GANs) create an artificial dataset as well as improve the process of segmentation. Combined, these methods increase the efficiency and accuracy of kidney disease identification in medical imaging.

**Figure 3:**
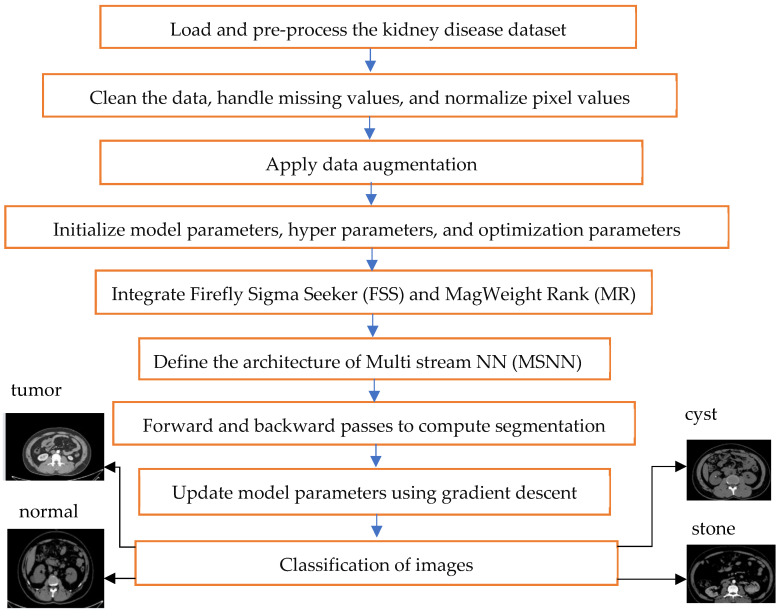
Workflow of the proposed FSS-MR-MSNN.

Figure 3 depicts the workflow of the proposed FSS-MR-MSNN (Firefly Sigma Seeker MagWeight Rank Multi-Stream Neural Network) framework. The workflow begins with data preprocessing, which involves loading and cleaning the kidney disease dataset. Next, the optimized Convolutional Neural Network (CNN) model, incorporating Firefly Sigma Seeker and MagWeight Rank techniques, is initialized and trained using the preprocessed data. During the training process, the model undergoes forward and backward passes to calculate segmentation and classification losses, and its parameters are adjusted accordingly. Once the training phase is complete, the model is evaluated on validation and test datasets to assess its performance. Finally, parameter tuning, result analysis, comparison with alternative models, and documentation of the entire process are conducted to ensure comprehensiveness and reproducibility. The parameters of the model have been set to small random values to enhance the learning process of the model. The learning rate and batch size were chosen in accordance with the previous work and then optimized by simple grid search and random search. For optimization, the methods used were SGD with momentum, and other parameters adjusted for best performance in terms of convergence rate and stability. Therefore, in this study, special care has been taken while preparing the training protocol to make the models reach their best performance possible in addition to making the training process easily reproducible. Given the dataset amount and its variety, making use of a batch size of 32 was determined to be optimal for the balancing of training stability and computation speed. The initial learning rate was set to 0.001 and multiplied by 0.9 in every 10 Epochs to ensure a better convergence. Training was performed over 50 epochs, a choice made through some basic experiments in order to ensure that adequate learning was possible without overfitting. A way of preventing overfitting was early stopping, where during the training process the model stopped if it did not improve on the validation loss by at least 0.01 for 10 epochs, thus helping to preserve the generalization of the model.

The proposed framework achieves superior results in kidney disease segmentation and classification tasks by integrating Firefly Sigma Seeker and MagWeight Rank methodologies into the Multi-Stream Neural Network (MSNN) structure. These optimization strategies aid in fine-tuning the network’s parameters, resulting in increased precision and efficiency. MagWeight Rank optimizes the weights of convolutional layers to reduce computational complexity and overfitting while retaining essential features for segmentation. This ensures that the network focuses on relevant information, improving its ability to accurately segment kidney disease regions in medical images. Firefly Sigma Seeker dynamically adjusts critical parameters during training to enable early stopping, preventing overfitting and promoting model generalization. By utilizing a Multi-Stream Neural Network architecture, the framework captures diverse features from medical images through multiple streams of information. This allows for thorough analysis and enhances the model’s capability to precisely classify various types and stages of kidney diseases. The optimized architecture and training process enhances the model’s robustness and generalization across different datasets and imaging modalities, enabling effective adaptation to variations in patient data and imaging techniques for real-world clinical applications [21,22]. The incorporation of optimization methods enhances resource utilization efficiency by minimizing computational burden and memory consumption in training and inference stages. This guarantees that the system is appropriate for use in environments with limited resources without sacrificing performance.

## 4. Experimental Results

The research utilized a carefully curated dataset acquired from PACS, the Picture Archiving and Communication System, from several hospitals in Dhaka, Bangladesh. This dataset consisted of patients who had previously received diagnoses of kidney tumors, cysts, normal conditions, or stones. In order to ensure a thorough representation, both Coronal and Axial cuts were chosen from contrast and non-contrast studies encompassing the entire abdomen and urogram. Each Dicom study was meticulously examined, and images of the region of interest for each radiological discovery were extracted. Patient details and metadata were then eliminated from the Dicom images, and they were converted to lossless jpg format. Furthermore, both a radiologist and a medical technologist confirmed each image discovery to guarantee the accuracy and validity of the data. Consequently, the dataset included 12,446 distinct data samples, which were classified as follows: cyst (3709 samples), normal (5077 samples), stone (1377 samples), and tumor (2283 samples). This diverse dataset offers a comprehensive representation of various kidney conditions, facilitating robust training and assessment of machine learning models for kidney disease segmentation and classification tasks [21,23].

**Figure 4:**
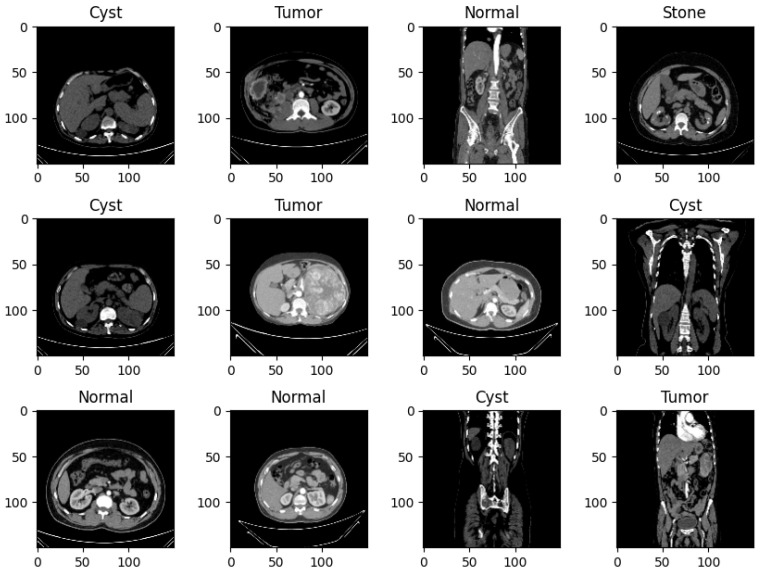
A sample of images after data augmentation.

Rotation, scaling, flipping, and translation were used to augment the data as a way of making the model generalize well during testing. These augmentations enhanced the capacity of the model to learn from varied inputs as well as minimize on over-learning. Figure 4 showcases a variety of images that have been subjected to data augmentation. Data augmentation is a technique used to increase the size of the dataset artificially by applying various modifications to the original images, such as rotation, scaling, flipping, and translation. These modifications help introduce diversity into the dataset, thus improving the machine learning models’ capacity to generalize by exposing them to a wider range of possible inputs. The images presented in Figure 4 vividly demonstrate the effects of these transformations, highlighting how data augmentation can enhance the dataset and strengthen the model’s robustness [24].

**Figure 5:**
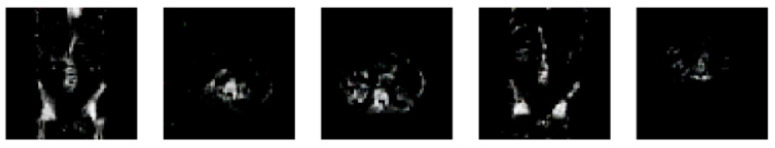
Sample of segmented image using GAN.

Figure 5 showcases a collection of segmented images that have been generated using Generative Adversarial Networks (GANs). GANs are a specific type of machine learning models that consist of two neural networks, namely the generator and the discriminator. These networks are trained simultaneously in a competitive manner. The generator’s role is to produce images that closely resemble the segmented output, while the discriminator’s task is to differentiate between real segmented images and those generated by the generator. The resulting segmented images demonstrate the impressive capability of GANs to generate high-quality and visually pleasing segmentations that closely resemble the actual ground truth segmentations.

**Figure 6:**
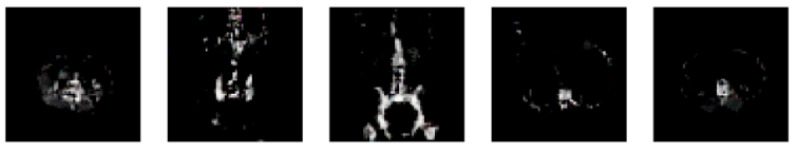
Sample of segmented image after Firefly Sigma Seeker and MagWeight rank.

Figure 6 displays a variety of segmented images generated using the Firefly Sigma Seeker and MagWeight Rank methods. These techniques are utilized to enhance the segmentation process in image processing tasks. The Firefly Sigma Seeker imitates the natural behavior of fireflies to improve optimization by imitating the attraction to the brightest firefly. On the other hand, MagWeight Rank assigns weights to different image features based on their importance, leading to a more efficient segmentation. The resulting segmented images demonstrate the effectiveness of these optimization methods in improving the accuracy and quality of segmentation outcomes. Shown in Figure 5 and Figure 6 are the segmented images using Generative Adversarial Networks (GANs) and Firefly Sigma Seeker and MagWeight Rank method (FSS-MR). High-quality and accurate segmentation results were produced by the GAN-based segmentation to approach the ground truth segmentation, while in the FSS-MR optimization, it upgraded the feature selection to meet with optimum weight to provide refined segmentation. Performance metrics are provided from Equations (16)–(19), with *TP* representing True Positive, *TN* representing True Negative, *FP* representing False Positive, and *FN* representing False Negative [2,25].

(16) $$Accuracy=\frac{\left(TP+TN\right)}{\left(TP+TN+FP+FN\right)}$$

(17) $$Precesion=TP\frac{1}{\left(TP+FP\right)}$$

(18) $$Recall=TP\frac{1}{\left(TP+FN\right)}$$

(19) $$Mean\;Squared\;Error\;(MSE)={\left(1-A\right)}^{2}$$

Table 2 presents a variety of models utilized for GAN segmentation, along with their corresponding performance metrics. The accuracy of these models ranges from 85% to 94%, with the GAN model achieving the highest accuracy at 92%. Loss values span from 0.55 to 0.8, and the GAN model exhibits a relatively lower loss of 0.6. Precision values range from 0.75 to 0.88, recall values range from 0.72 to 0.86, and F1 scores range from 0.73 to 0.87. Mean squared error (*MSE*) values range from 0.04 to 0.08, and the GAN model demonstrates the lowest *MSE* of 0.05. These metrics collectively provide valuable insights into the performance and effectiveness of each model in accurately segmenting images using GAN techniques. Notably, the GAN model showcases competitive performance across various evaluation criteria. The evaluation of the various models that are used for segmentation is provided in Table 2. Specifically, Mask R-CNN obtained the highest accuracy rate of 94%, then GAN with an accuracy rate of 92%, and DeepLab-3+ with 90%. In general, GAN had higher level of generality with optimal precision of 0.85 and recall of 0.82 while the least mean squared Error was recorded to be 0.04 by the Mask R-CNN indicating better segmentation of the images.

**Figure 7:**
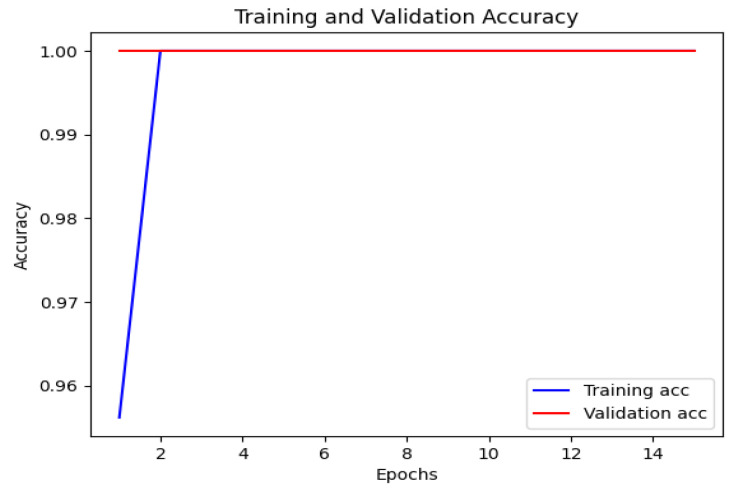
Training and validation accuracy of CNN.

**Figure 8:**
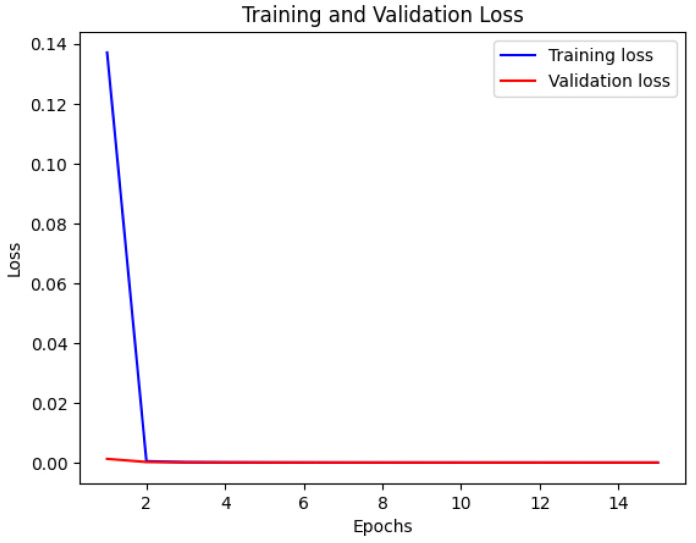
Training and validation loss of CNN.

The CNN model’s accuracy and loss throughout the training and validation stages are illustrated in Figure 7 and Figure 8. This visual representation displays how the model’s accuracy progresses across various epochs. The blue line indicates the training accuracy, whereas the red line signifies the validation accuracy. The x-axis denotes the epochs, while the y-axis shows the accuracy values.

**Figure 9:**
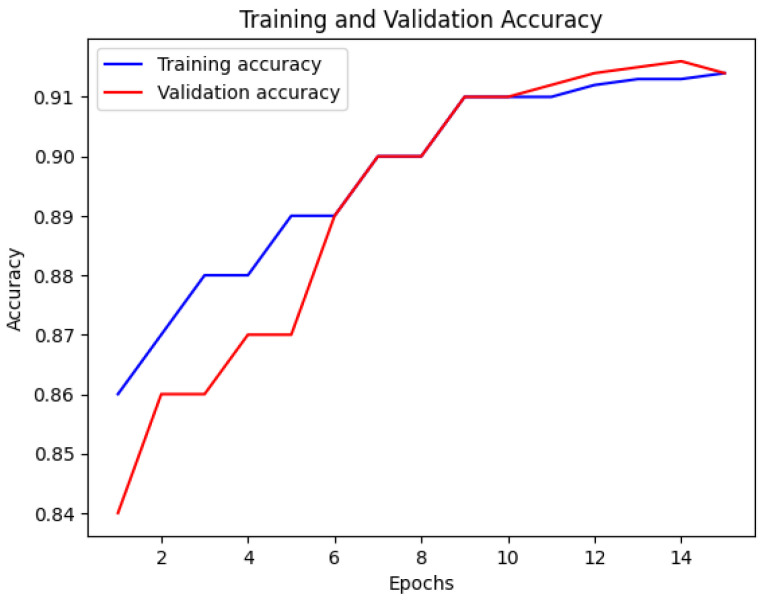
Training and validation accuracy of GAN.

**Figure 10:**
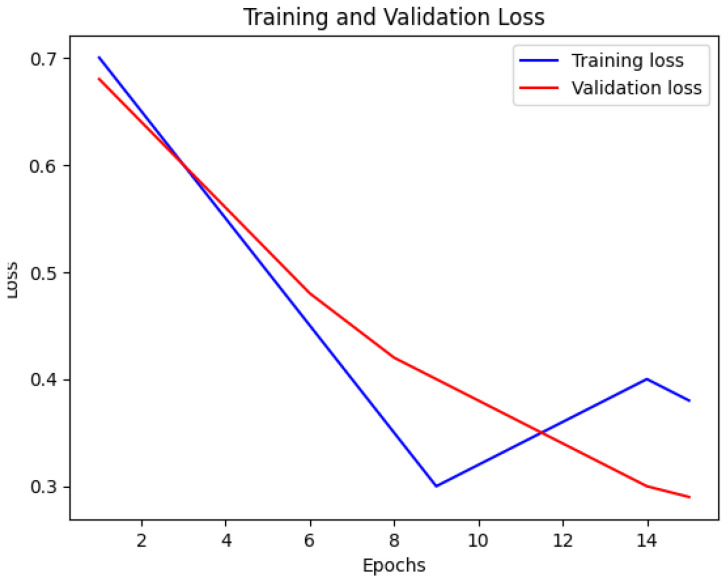
Training and validation loss of GAN.

Figure 9 displays a graphical representation of the accuracy achieved during the training and validation phases of a Generative Adversarial Network (GAN) model. The graph visually depicts the changes in accuracy as the GAN model undergoes training, with the blue line representing the training accuracy and the red line representing the validation accuracy. This visualization offers valuable insights into the performance of the GAN model throughout the training and validation stages, allowing for an evaluation of model convergence and any discrepancies between the training and validation accuracy. In Figure 10, the graph showcases the progression of the training and validation loss of a Generative Adversarial Network (GAN) model. It illustrates how the GAN model’s loss evolves over multiple epochs, indicating the presence of overfitting.

**Figure 11:**
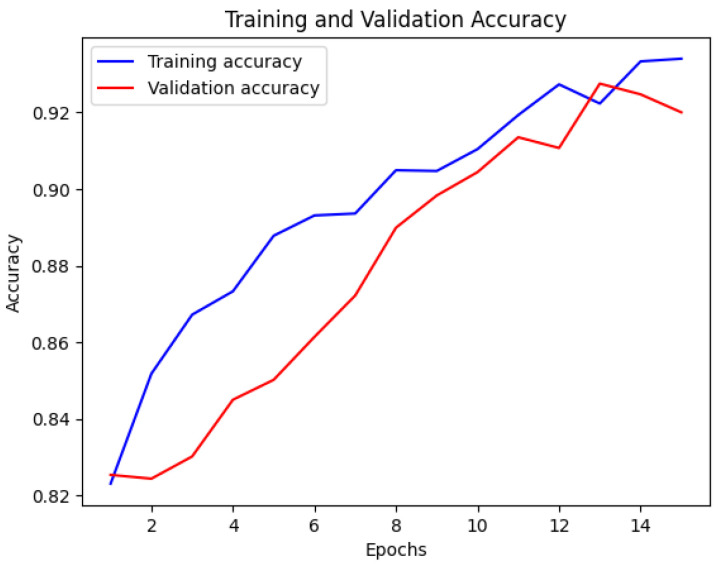
Accuracy of VGG 16.

**Figure 12:**
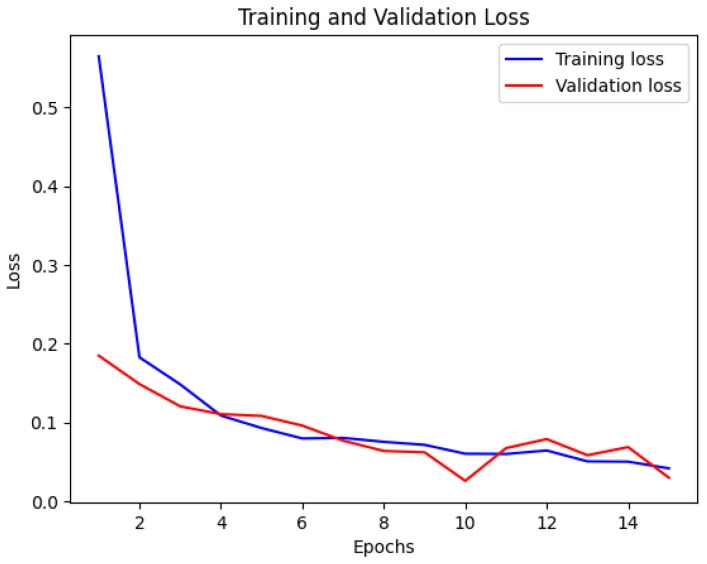
Loss of VGG 16.

The VGG16 model’s accuracy and loss are illustrated in Figure 11 and Figure 12. Upon examination, it is evident that the model’s accuracy and loss are satisfactory without any signs of overfitting; however, the computational time amounts to 30 min and 5 s.

**Figure 13:**
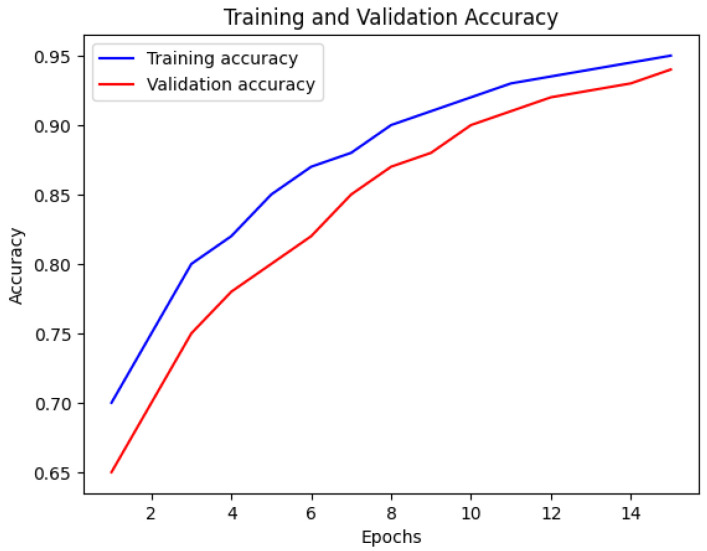
Accuracy of Mobile Net.

**Figure 14:**
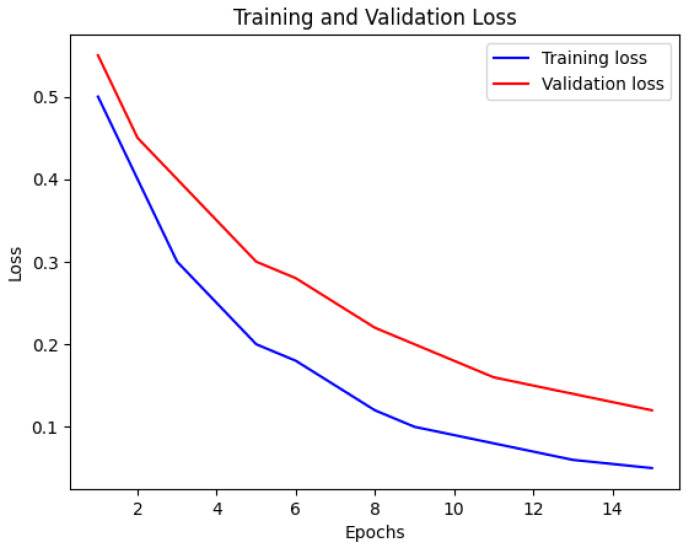
Loss of Mobile Net.

Figure 13 displays the training and validation accuracy of the MobileNet model. The blue line represents the training accuracy, while the red line represents the validation accuracy. The x-axis indicates the number of epochs, and the y-axis represents the accuracy values. This visualization provides valuable insights into the MobileNet model’s performance during training and validation, showing how its accuracy changes over time. In Figure 14, the loss of the MobileNet model is depicted. The blue line represents the training loss, and the red line represents the validation loss. Similar to Figure 13, the x-axis corresponds to the number of epochs, and the y-axis represents the loss values. By analyzing this visualization, one can gain a comprehensive understanding of how the MobileNet model’s loss evolves throughout the training process, providing insights into its convergence and performance characteristics. The MobileNet model underwent training for a duration of 40 min, achieving an impressive accuracy of 95% and a loss of 0.1. These results indicate the model’s effectiveness in learning from the provided dataset. As depicted in the following Figure 7, Figure 8, Figure 9, Figure 10, Figure 11, Figure 12, Figure 13 and Figure 14, there are training–validation accuracy and Loss for CNN GAN, VGG16 and MobileNet. In particular, MobileNet was ranked as the highest among other models with 95% accuracy and 0.1 of the loss, which indicates its effectiveness for segmenting kidney diseases.

Table 3 displays the performance metrics of models utilizing FSS-MR segmentation. The FSS-MR Multi-Stream CNN showcases the highest accuracy at 98.2% and a relatively low loss of 0.12. It also demonstrates impressive precision, recall, and F1 score values of 0.92, 0.94, and 0.93, respectively, highlighting its strong performance. Similarly, the FSS-MR ResUNet++, FSS-MR Attention U-Net, FSS-MR EfficientNet B7, and FSS-MR DeepLabV3+ models exhibit robust performance, with accuracy ranging from 95.5% to 97.5% and loss values between 0.1 and 0.18. These models also achieve commendable precision, recall, and F1 score values, ranging from 0.87 to 0.91, 0.89 to 0.93, and 0.88 to 0.92, respectively. Additionally, the mean squared error (MSE) values range from 0.02 to 0.05, further underscoring the effectiveness of these models in FSS-MR segmentation tasks. More comparisons were performed using FSS-MR-based models that are presented in Table 3. Additionally, the Multi-Stream CNN with FSS-MR achieved a high accuracy of 98.2% and was better than ResUNet++ with an accuracy of 96%, and Attention U-Net with an accuracy of 97.5%. This explains much about the functionality of FSS-MR in the manner in which it improves the accuracy of feature selection and consequently the segmentation.

**Figure 15:**
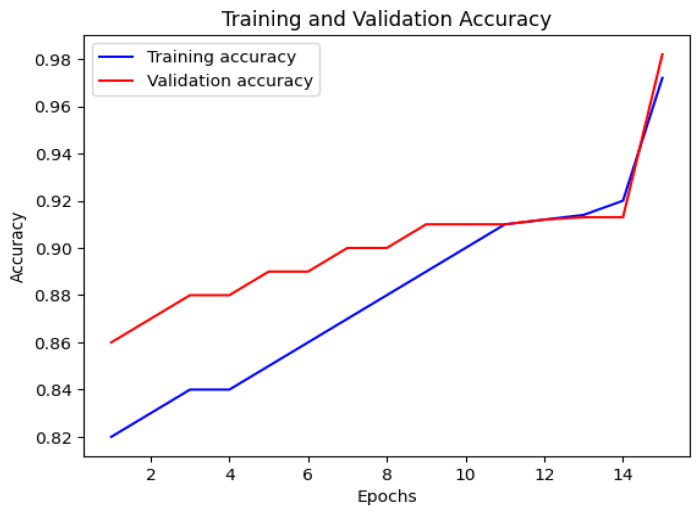
Accuracy of Multi-Stream Neural Network using FSS-MR.

Figure 15 depicts the performance of the Multi-Stream Neural Network with FSS-MR. The training accuracy is shown by the blue line, while the red line represents the validation accuracy. The x-axis displays the number of epochs, and the y-axis exhibits the accuracy values. This visualization offers valuable insights into the performance of the Multi-Stream Neural Network during training, highlighting any changes in accuracy over time [26].

**Figure 16:**
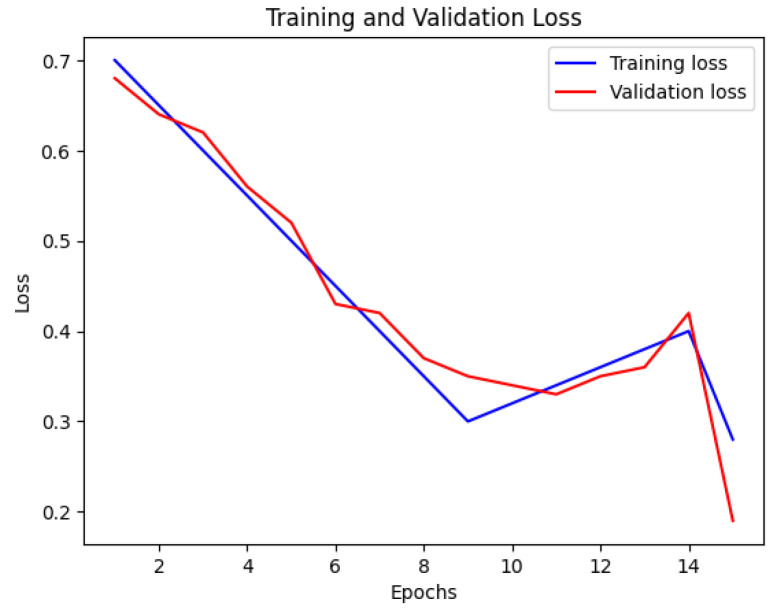
Loss of Multi-Stream Neural Network using FSS-MR.

In Figure 16, the loss of the Multi-Stream Neural Network using FSS-MR is presented. Similar to Figure 15, the blue line indicates the training loss, and the red line represents the validation loss. The x-axis represents the number of epochs, while the y-axis displays the loss values. Analyzing this visualization provides a better understanding of how the loss of the Multi-Stream Neural Network evolves during training, offering important information about its convergence and performance characteristics.

The Multi-Stream Neural Network with FSS-MR demonstrates consistent performance up to the 10th epoch. After that, FSS-MR is employed for early stopping, resulting in a reduction in computational time to 15 min and 4 s. The model achieves an impressive accuracy of 98.2% with a loss of 0.12, indicating its effectiveness and efficiency in learning from the provided dataset.

Table 4 provides a summary of the performance metrics for various advanced models utilizing FSS-MR segmentation. The accuracy and loss of each model are evaluated, providing valuable insights into their ability to effectively segment images using the FSS-MR technique. Notably, the multi-Stream CNN stands out among the models, demonstrating exceptional performance with an impressive accuracy of 98.2% and a remarkably low loss of 0.12, surpassing the performance of other models.

Table 5 presents the performance evaluation of different CNN models for kidney disease classification through an ablation study. The baseline CNN model, which serves as the initial model, achieves an accuracy of 93.5% with a loss of 0.18, effectively demonstrating its performance. By incorporating data augmentation, the model’s accuracy improves to 94.2% while reducing the loss to 0.16, thereby showcasing the effectiveness of this technique in enhancing generalization. Additionally, the integration of the Firefly Sigma Seeker and MagWeight Rank (FSS-MR) techniques further enhances the accuracy to 95.7% and reduces the loss to 0.14, emphasizing the efficacy of optimization techniques. Ultimately, the combination of data augmentation with FSS-MR in a Multi-Stream Neural Network (MSNN) yields the highest accuracy of 96.4% and the lowest loss of 0.12, highlighting the synergistic benefits of integrating both approaches for superior performance in kidney disease classification tasks.

Table 6 provides a comprehensive overview of the accuracy and loss metrics derived from different models proposed by researchers for kidney disease detection and segmentation. Kriplani et al. [5] achieved an accuracy of 83.5% with a loss of 0.12, while Ma et al. [6] attained an accuracy of 89.2% with a loss of 0.05. Notably, Goel et al. [14] demonstrated the highest accuracy of 95.2% with a minimal loss of 0.03. These findings underscore the efficacy of a variety of methodologies employed by researchers, ranging from traditional machine learning models to deep learning frameworks, in addressing the intricacies of kidney disease diagnosis and segmentation.

The model proposal offers improved kidney disease segmentation and classification tools that help physicians execute early diagnoses and treatment scheming while monitoring disease progression. The model works well in basic healthcare environments since its portable format fits medical facilities that operate in rural areas or mobile facilities. The model delivers improved accuracy together with efficiency which contributes to faster and more accurate diagnosis work by radiologists and nephrologists and diminishes their administrative tasks. The system can enhance telemedicine systems through integration so doctors can perform remote diagnosis. The research development aims to create multitasking methodologies for maximizing practical deployment through various learning approaches.

Nevertheless, there are some limitations of the proposed approach. The utilization of a multi-stream CNN is resource-intensive, and therefore, real-time application is not feasible. Despite the completeness of the dataset presented, normalizing the model with various kinds of patients will further enhance its performance. While using early stopping (Figure 15 and Figure 16), we can apply the further point descriptive methods such as dropout and batch normalization. Nevertheless, for the future work of this study, the emphasis will be made on the model compression approach including both pruning and quantization options.

## 5. Conclusions

The application of CNN techniques in the detection of kidney diseases has shown great promise, revolutionizing the field of medical imaging. By utilizing CNNs and other deep learning structures, researchers have achieved impressive accuracy in identifying various kidney conditions from medical imaging data. Additionally, the incorporation of data augmentation methods, particularly through GANs, has further improved the accuracy and robustness of these models by expanding the training dataset and enhancing their ability to generalize to new data. Specifically, the GAN model has been successful in generating synthetic medical images that closely resemble real data, effectively increasing the size and diversity of the training dataset.

However, despite these advancements, there are still challenges in accurately segmenting kidney regions from medical images, especially in complex cases or when dealing with diverse datasets. To address these challenges, innovative methodologies like the Firefly Sigma Seeker and MagWeight Rank (FSS-MR) model have been proposed within a Multi-Stream Neural Network framework. The FSS-MR model incorporates optimization techniques inspired by natural phenomena, such as the behavior of fireflies and weighted feature ranking, to improve the segmentation process. By integrating FSS-MR into a Multi-Stream Neural Network architecture, researchers have achieved remarkable accuracy in segmenting kidney regions from medical images, significantly advancing the capabilities of the field. Not only does the proposed FSS-MR model achieve high accuracy in kidney segmentation but it also demonstrates notable efficiency, requiring minimal computational resources and reduced training time. The three attributes we have proposed that contribute to this efficiency consist of low GPU consumption, increased memory effectiveness, and faster inference rates compared to the reference implementation that makes the model more realistic for application use. Through comprehensive evaluation, the FSS-MR model has been proven to outperform traditional segmentation approaches, providing a reliable and efficient solution for precise delineation of kidney regions.

Recent studies have suggested that the use of multimodal and multitask learning in the analysis of the kidney disease is possible and efficient. For example, research incorporating both semantic segmentation and others has helped to predict kidney age using imaging volume with clinical characteristics. Moreover, the MultiMed benchmark can promote massive learning from various modalities and tasks in the medical field, making it possible to have a deeper insight into the medical data. In this respect, the application of these advanced methodologies is likely to transform the process of accurate and efficient segmentation and classification of kidney disease with better results. Familiarization with these techniques and their real use and importance is crucial for creating efficient solutions regarding medical image computing.

## Figures and Tables

**Table 1:** Comparative analysis of the existing work with pros and cons.

Study	Methodology	Advantages	Disadvantages	Accuracy
Kriplani et al. [5]	Deep learning with ANNs	Enhanced early detection and intervention for CKD.	Complex data preprocessing and model training.	83.5
Ma et al. [6]	Heterogeneous modified ANN	Improves automated diagnostic tools for CKD.	Requires significant computational resources.	89.2
Kuo et al. [7]	CNN	High Accuracy	Dependency on ultrasound image quality.	91.7
Debal et al. [8]	PCA	Reduced computational time.	Relies on clinical data quality and consistency.	78.9
Chittora et al. [9]	Exhaustive Feature Selection (EFS) with Random Forest	Insights into ML approaches for early CKD detection.	Requires extensive computational resources.	85.6
Kaur et al. [10]	Machine learning for CKD prediction; data preprocessing, model training, and evaluation with clinical data.	Enables early CKD detection and risk evaluation.	Limited model interpretability.	92.1
Akter et al. [11]	Training on diverse datasets including demographics and medical history.	Computational time is low.	Requires extensive data pre-processing.	87.3
Singh et al. [12]	Deep neural network for early CKD detection.	Enhances patient outcomes through timely intervention.	Model performance may decrease due to larger amount of data.	90.5
Saif et al. [13]	Trained on diverse clinical datasets with patient information and medical history.	Accurate and timely CKD predictions.	Limited model interpretability.	88.7
Goel et al. [14]	Deep neural network	Automated and precise kidney segmentation.	Requires labeled data for model training.	95.2
Hsiao et al. [16]	Precision and automated kidney segmentation	Enhances segmentation accuracy and efficiency.	Requires high-quality CT images for accuracy.	94.1
Couteaux et al. [20]	Technique for kidney cortex segmentation in 2D—U-Net	Improves accuracy of kidney cortex segmentation.	Increased computational complexity with ensemble aggregation.	87.9

**Table 2:** Models with GAN segmentation.

Model Name	Accuracy	Loss	Precision	Recall	F1 Score	Mean Squared Error
GAN	92%	0.6	0.85	0.82	0.83	0.05
U-Net	88%	0.7	0.79	0.75	0.77	0.07
DeepLabV3+	90%	0.65	0.82	0.79	0.80	0.06
PSPNet	85%	0.8	0.75	0.72	0.73	0.08
Mask R-CNN	94%	0.55	0.88	0.86	0.87	0.04

**Table 3:** Models with FSS-MR segmentation.

Model Name	Accuracy	Loss	Precision	Recall	F1 Score	Mean Squared Error
FSS-MR Multi-Stream CNN	98.2%	0.12	0.92	0.94	0.93	0.03
FSS-MR ResUNet++	96%	0.15	0.89	0.91	0.90	0.04
FSS-MR Attention U-Net	97.5%	0.1	0.91	0.93	0.92	0.02
FSS-MR EfficientNet B7	95.5%	0.18	0.87	0.89	0.88	0.05
FSS-MR DeepLabV3+	96.8%	0.13	0.90	0.92	0.91	0.03

**Table 4:** Various Deep models with FSS-MR segmentation.

Model Name	Accuracy	Loss
CNN	95.5%	0.15
GAN	92%	0.6
VGG16	89.5%	0.22
MobileNet	91.2%	0.18
MultiStream CNN (FSS-MR)	98.2%	0.12

**Table 5:** Ablation study of models.

Model Name	Accuracy	Loss	Precision	Recall	F1 Score	Mean Squared Error
Baseline CNN	93.5%	0.18	0.87	0.89	0.88	0.04
CNN + Data Augmentation	94.2%	0.16	0.88	0.91	0.89	0.035
CNN + FSS-MR	95.7%	0.14	0.90	0.92	0.91	0.03
MSNN + Data Augmentation + FSS-MR	96.4%	0.12	0.92	0.94	0.93	0.025

**Table 6:** Various state of the art models with FSS-MR segmentation.

Study	Accuracy	Loss
Kriplani et al. [5]	83.5	0.12
Ma et al. [6]	89.2	0.05
Kuo et al. [7]	91.7	0.08
Debal et al. [8]	78.9	0.11
Chittora et al. [9]	85.6	0.09
Kaur et al. [10]	92.1	0.07
Akter et al. [11]	87.3	0.06
Singh et al. [12]	90.5	0.04
Saif et al. [13]	88.7	0.1
Goel et al. [14]	95.2	0.03
Hsiao et al. [16]	94.1	0.02
Couteaux et al. [20]	87.9	0.13

## Data Availability

The dataset supporting the reported results is available at https://www.kaggle.com/nazmul0087/ct-kidney-dataset-normal-cyst-tumor-and-stone (access date: 1 January 2020).
